# Assessment of the Genetic Diversity of the Monogenean Gill Parasite *Lamellodiscus echeneis* (Monogenea) Infecting Wild and Cage-Reared Populations of *Sparus aurata* (Teleostei) from the Mediterranean Sea

**DOI:** 10.3390/ani14182653

**Published:** 2024-09-12

**Authors:** Sarra Farjallah, Nabil Amor, Francisco Esteban Montero, Aigües Repullés-Albelda, Mar Villar-Torres, Abdulaziz Nasser Alagaili, Paolo Merella

**Affiliations:** 1 Laboratory of Ecology, Biology and Physiology of Aquatic Organisms LR18ES41, Faculty of Sciences of Tunis, University of Tunis El Manar, Tunis 2092, Tunisia; nabil.amor@issbat.utm.tn; 2 Marine Zoology Unit, Cavanilles Institute of Biodiversity and Evolutionary Biology, Science Park, University of Valencia, C/Catedrático José, Beltrán 2, 46980 Paterna, Spain; francisco.e.montero@uv.es (F.E.M.); aigues.repulles@uv.es (A.R.-A.); mar.villar@uv.es (M.V.-T.); 3 Department of Zoology, King Saud University, P.O. Box 2455, Riyadh 11451, Saudi Arabia; ziz99@gmail.com; 4 Department of Veterinary Medicine, University of Sassari, Via Vienna, 2, 07100 Sassari, Italy; paolomerella@uniss.it

**Keywords:** *Lamellodiscus echeneis*, *Sparus aurata*, population genetics, phylogenetic, gene flow, population expansion, cross-infection

## Abstract

**Simple Summary:**

This research represents the first analysis of the population genetics of the diplectanid monogenean *Lamellodiscus echeneis*, sampled from wild and cage-reared gilthead seabream *Sparus aurata* across fifteen locations in the Southern (Tunisia) and Northern (Italy and Spain) regions of the Mediterranean Sea. The comparison of the newly obtained dataset to previously published sequences of *L. echeneis*, and the phylogenetic trees based on the analysis of ITS rDNA, confirmed the presence of only one species in the Mediterranean Sea. The star-like haplotype network inferred by COI sequences suggested a recent population expansion of *L. echeneis*. This study provides insights into the genetic variation among *L. echeneis* populations and did not reveal a clear genetic structure throughout Tunisian, Italian, and Spanish localities, possibly due to significant gene flow between the populations facilitated by the dispersion potential of hosts.

**Abstract:**

The diplectanid monogenean *Lamellodiscus echeneis* (Wagener, 1857) is a specific and common gill parasite of the gilthead seabream *Sparus aurata* Linnaeus, 1758, in the Mediterranean Sea. Few isolated molecular studies of this monogenean have been conducted, and its population structure and genetic diversity are poorly understood. This study represents the first analysis of the population genetics of *L. echeneis*, isolated from wild and cage-reared gilthead seabream from fifteen localities in both the Southern (Tunisia) and Northern (Italy and Spain) regions of the Mediterranean Sea, using nuclear ITS rDNA markers and a partial fragment of the mitochondrial gene cytochrome oxidase subunit I (COI). The phylogenetic trees based on the newly obtained dataset and the previously published sequences of *L. echeneis* corroborated the spread of only a single species throughout the Mediterranean Sea. The star-like haplotypes network, inferred by COI sequences, suggested a recent population expansion of *L. echeneis*. This is supported by the observed high haplotype diversity (Hd = 0.918) and low nucleotide diversity (Pi = 0.01595). Population structure-based AMOVA for two groups (the Adriatic Sea and the rest of the Mediterranean Sea) attributed 35.39% of the total variation to differences within populations, 16.63% to differences among populations within groups, and 47.99% to differences among groups. Fixation indices were significant, with a high FST value (0.64612), likely related to the divergence of the parasite populations from the Adriatic Sea and other Mediterranean regions. Phylogenetic analyses grouped all samples into the main clade corresponding to *L. echeneis* from several localities. This study provides insight into the genetic variation between *L. echeneis* populations, and did not show a clear genetic structure between populations of *L. echeneis* throughout Tunisian, Italian, and Spanish localities, which can be attributed to the considerable gene flow between the populations favoured by the potential for host dispersion within the Mediterranean Sea. Finally, haplotypes shared between wild and cage-reared hosts provided evidence for the potential for cross-infection between wild and farmed hosts in the Mediterranean Sea.

## 1. Introduction

Aquaculture in the Mediterranean Sea has become essential in the food sector, due to the decline of wild fish stocks, and is recognised as a major contributor to seafood for human consumption. The gilthead seabream *Sparus aurata* Linnaeus, 1758, is a common fish species throughout the Mediterranean Sea and the Eastern Atlantic Ocean; it is considered one of the most popular breams for food. As a fish species of significant economic value, gilthead seabream production reached 143,106 tons in 2012, and has more than doubled to 344,094 tons in 2022, with Turkey, Egypt, Tunisia, and Saudi Arabia representing more than two-thirds of the total production, and Greece, Spain, Italy, and Croatia as major producers in Europe [1]. Monogeneans are one of the largest groups of Platyhelminthes, generally inhabiting the skin and gills of fish, characterised by a relatively high degree of host-specificity and a direct life cycle [2]. Among them, diplectanids present numerous attachment mechanisms due to differences in the haptor structure, leading to several detrimental effects and damage on host gill tissues [3,4,5]. The diplectanid monogenean *Lamellodiscus echeneis* (Wagener, 1857) (syn. *Furnestinia echeneis*) is a specific and common gill parasite of wild and cultured gilthead seabreams in the Mediterranean Sea. According to Sánchez-García et al. [5], the hypertrophied ventral lamellodisc of *L. echeneis* works as a suction organ for the attachment to the primary lamellae, and this attachment strategy seems not to affect the respiratory structures, causing only mild damage, in contrast to other diplectanids [4]. This species was previously included in the monospecific genus *Furnestinia,* based on the presence of a single adhesive organ on the haptor [6,7,8]; however, a phylogenetic study of the family Diplectanidae showed that the *Furnestinia* and *Lamellodiscus* genus should be merged together [8]. Numerous studies have employed genetic markers for molecular systematics and species identification within monogeneans [9,10,11,12,13,14,15]. In molecular systematics, the nuclear ITS regions have been effectively used for phylogenetic analysis and species differentiation due to their significant degree of sequence variation. Many studies have confirmed the applicability of ITS rDNA regions to identify monogeneans for diagnostic purposes [12,16]. Mitochondrial DNA genes have also been employed to differentiate between monogenean species and estimate their intraspecific genetic variations, as shown in studies involving Gyrodactylidae, Microcotylidae and Diplectanidae [17,18,19,20,21,22,23]. In addition, many molecular studies have shown that a multilocus approach to assess phylogenetic relationships within organisms provides more consistent results [22,24,25,26,27,28,29,30]. Despite the significant prevalence of *L. echeneis* on the gills of wild and reared gilthead seabream, and its widespread distribution in the Mediterranean Sea [31,32], only a few isolated molecular studies of this species have been conducted on wild and cultured hosts [8,33], and its population structure and genetic diversity are poorly known. In the present study, the population genetic variation of *L. echeneis* isolated from cage-reared and wild *S. aurata* from several Mediterranean localities off of Tunisia, Italy, and Spain is evaluated using nuclear ITS rDNA and partial mitochondrial cytochrome oxidase I (COI) markers. The population genetic structure of *L. echeneis* in the Mediterranean Sea is analysed by integrating new and previously published haplotype sequences, and its phylogenetic status is described.

## 2. Materials and Methods

### 2.1. Collection of Hosts and Parasitological Analysis

**Figure 1:**
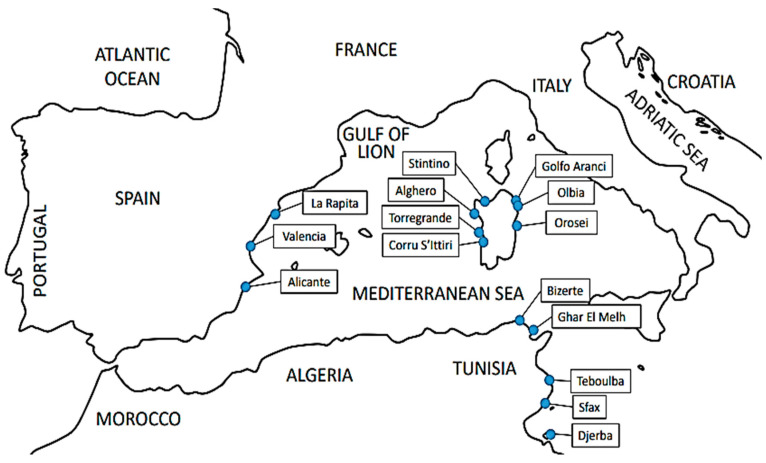
Map of the Mediterranean localities where specimens of *Sparus aurata* infected with *Lamellodiscus echeneis* were collected.

Specimens of *L. echeneis* were collected on the gills of 160 specimens of *S. aurata* from different localities of Tunisia, Italy, and Spain (Figure 1, Table 1). In particular, between March and August 2023, specimens of wild (*n* = 20) and cage-reared (*n* = 30) gilthead seabream were collected from five localities of Tunisia; between 2005 and 2023, wild (*n* = 14) and cage-reared (*n* = 30) gilthead seabream were collected from six localities of Sardinia (Italy); and in 2023, wild (51) and cage-reared (15) gilthead seabreams were collected from three Mediterranean Spanish localities (Figure 1). Specimens of *L. echeneis* were preserved in 99% ethanol. The number of specimens included in the molecular analysis, along with collection localities and dates considered in this study, are shown in Table 1.

### 2.2. DNA Extraction, Amplification, and Sequencing

Genomic DNA was extracted from *L. echeneis* samples, representing fifteen localities from three countries in the Mediterranean Sea, as described by Farjallah et al. [22] (Table 1). Two molecular markers were used for monogeneans haplotyping, as follows: partial 18S, complete ITS1, and partial 5.8S (ITS) of the rDNA and the cytochrome C oxidase subunit 1 (COI) of the mtDNA. For the phylogenetic analysis, the partial fragment of the ITS ribosomal DNA gene was amplified using the primers ITSfurF (5′-*CATCGTCGTGCCTGGGA*-3′) and ITSfurR (5′-*GTACATAGACATCACACCAAGGT*-3′) (Table 1) [33]. To explore the population genetic variation, a second set of primers, COIfurF (5′-*GAGCTAAGTAAAAATCAAGAACC*-3′) and COIfurR (5′-*TCTATCTAACACTGA GGCTG*-3′), was employed for the amplification of 266 bp fragments of the cytochrome oxidase I (COI) (Table 1) [33]. Each amplification was performed as described by Mladineo et al. [33]. The amplified products were examined by gel electrophoresis (1% agarose), with the molecular weight marker HyperLadder 100 bp (Bioline Reagents Ltd., London, UK). PCR products were sequenced at Macrogen (Macrogen Inc., Amsterdam, The Netherlands, Europe). The obtained sequences were manually checked and aligned using CLUSTALW, implemented in Mega X version 10.2.5 [34]. Sequence alignments included available sequences in GenBank obtained using BLAST algorithm [35]. Comparative sequence analyses were conducted using available ribosomal and mitochondrial sequences of *L*. *echeneis* from the Adriatic Sea (*n* = 51; 13 wild and 38 cage-reared gilthead seabream) and the Gulf of Lion (*n* = 3; wild gilthead seabream) [33] (Supplementary Materials Tables S1 and S2). The hosts, sources, and accession numbers of ITS and COI sequences are presented in Tables S1 and S2 of the Supplementary Materials. The sequence of *Dolicirroplectanum lacustre* (MK908196) was applied as outgroup for the COI analysis, although sequences of *Pseudorhabdosynochus lantauensis* (AY553614), *Diplectanum blairense* (DQ537356), *D. sillagonum* (AY553617), and *Lamellodiscus* sp. were used for the ITS tree rooting.

### 2.3. Population Genetic Analyses

DnaSP v.5.10.01 [36] was used to estimate the number of haplotypes (H), haplotype diversity (Hd), nucleotide diversity expressed as the average number of nucleotide differences between two sequences by site (Pi), the average number of nucleotide differences between sequences (k), the number of polymorphisms and insertions/deletions (S), and pairwise FST. The gene flow estimate, Nm, was calculated as suggested by Hudson et al. [37] for the mitochondrial nucleotide sequence data information, using the formula Nm = ½(Hw/(Hb − Hw)).

Haplotype networks, based on the mitochondrial molecular marker, were generated using PopArt [38] to depict relationships among *L*. *echeneis* populations from different geographical regions. The median-joining (MJ) network algorithm was used with default parameters (equal character weight 10, transitions/transversions weight 1:1, and connection cost as a criterion).

Analysis of molecular variance (AMOVA) was assessed among *L. echeneis* populations using Arlequin 3.5 [39]. *Lamellodiscus echeneis* samples were grouped according to their geographic origin into five groups (Tunisia/*S. aurata*, Italy/*S. aurata*, Spain/*S. aurata*, Gulf of Lion/*S. aurata*, Adriatic Sea/*S. aurata*). A second AMOVA was performed, including *L. echeneis* samples isolated from the Adriatic Sea and the remaining Mediterranean localities (Adriatic Sea/*S. aurata*, Mediterranean localities/*S. aurata*). A last AMOVA was performed, including all *L. echeneis* samples, except for those from the Adriatic Sea, grouped into four groups. Pairwise genetic distances between the studied localities (p-distance values) were evaluated using Mega X version 10.2.5 [34]. To confirm whether expansion occurred, mismatch distributions (frequency of pairwise nucleotide-site differences between sequences) were assessed using Arlequin 3.5 [39].

### 2.4. Phylogenetic Analysis

The appropriate models of evolution and the partitioning scheme were inferred using PartitionFinder 2.1 [40]. For both mitochondrial and nuclear markers, Neighbour-Joining (NJ) and Maximum Likelihood (ML) phylogenetic trees were constructed using Mega X version 10.2.5 and RAXML version 8 [41]; bootstrap values were evaluated by 2000 pseudoreplicates. Bayesian (BI) analyses were conducted using MrBayes version 3.2.6 [42], with two independent runs of 1 × 10^8^ generations. The chains were sampled every 1000 generations.

## 3. Results

A total of ninety-seven partial COI and twenty-three ITS sequences were obtained for the specimens of *F. echeneis* from the fifteen localities of the Mediterranean Sea. Careful visual inspection of the chromatogram data showed no double peaks, ensuring that each chromatogram consistently displayed a single unambiguous peak. Furthermore, during the subsequent translation of the sequences, each coding region was systematically examined to identify the presence of any stop codons. Comprehensive scrutiny in both aspects did not reveal any instances of double peaks or stop codons, thus affirming the thorough validation and integrity of the data. A comparison of the *L. echeneis* ITS region of rDNA exhibited high blast scores, with previously published *L. echeneis* sequences in the GenBank. The highest genetic similarity was 100%, to specimens from the Gulf of Lion (AF294953), and 99%, to specimens from the Adriatic Sea (JX090055, JX090091, JX090095, JX090089, JX090086, JX090056, JX090048, JX090083) and the Gulf of Lion (JX090045, JX090048).

### 3.1. Population Genetic Analysis

The multiple sequence alignment of the partial COI (266 bp) included 38 new sequences from Tunisia, 32 from Italy, 27 from Spain, and 54 previously published sequences from the Adriatic Sea and the Gulf of Lion [33]. COI variation was mostly related to mutations at the third codon position. Alignment of the partial COI sequences contained 35 polymorphic sites and 19 parsimony-informative sites, defining a total of 57 haplotypes (44 haplotypes from Tunisia, Italy, and Spain). The obtained haplotypes were submitted in GenBank under the accession numbers PP892317-73. The mitochondrial COI marker was characterised by having high haplotype diversity (Hd = 0.918 ± 0.014 S.D.), although its nucleotide diversity was low (Pi = 0.01595 ± 0.00653 S.D.). The average number of nucleotide differences (k) was 4.242. Haplotype diversity was high in every *L. echeneis* population, ranging from 0.60157–1. Nucleotide diversity was low for each of these populations, ranging between 0.00373 and 0.01383 (Table 2).

**Figure 2:**
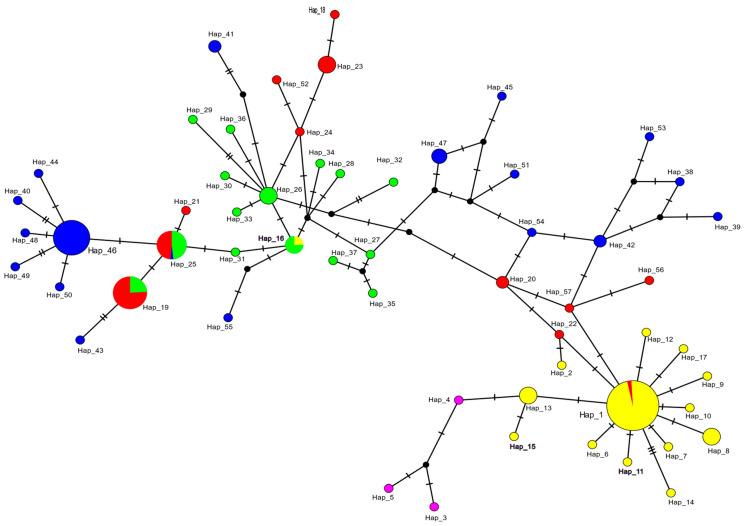
Haplotype network for *Lamellodiscus echeneis* from the gills of *Sparus aurata* from the Mediterranean Sea based on 266 bp COI sequences. The hatch marks represent the number of mutational steps between haplotypes. The black circles indicate alternative unsampled haplotypes. Pie chart sizes are proportional to haplotype frequency. Circle colours indicate the locations of origin for the distinct haplotypes. Yellow: The Adriatic Sea; pink: the Gulf of Lion; red: Sardinia, Italy; green: Spain; blue: Tunisia. Abbreviations: H1–H57, haplotype IDs.

In the COI network, most haplotypes were singletons (78.94%) and were represented by a unique individual (Figure 2). No haplotypes were shared across the complete species distribution range. The COI haplotype network revealed a “star-like” pattern, with the major haplotype, Hap_1, including thirty-two sequences from the Adriatic Sea and three from Italy (Torre Grande and Golfo Aranci); the latter originated from cage-reared *S. aurata* (Figure 2). From Hap_1 radiated a crown of less common haplotypes, including twelve haplotypes from the Adriatic Sea (Hap_2, Hap_6–15, and Hap_17) and four from Italy (Hap_20, Hap_22, and Hap_56–57), separated by one to three mutation steps (Figure 2). The second most common haplotype, Hap_46, included 17 sequences from both wild (Sfax and Bizerte) and cage-reared (Ghar El Melh, Djerba, and Teboulba) gilthead seabream in Tunisia (Figure 2). This haplotype clustered with six other haplotypes (Hap_25, Hap_40, Hap_44, and Hap_48–50), separated by one to two mutation steps. The haplotype Hap_26 included four Spanish samples from cage-reared (Alicante) and wild (Sant Carles de la Rapita and Valencia) hosts, grouped with one haplotype from Tunisia (Hap_41), four haplotypes from Italy (Hap_18, Hap_23–24, and Hap_52), and four from Spain (Hap_29, Hap_30, Hap_33, and Hap_36). Hap_26 was connected to haplotype Hap_16, including three sequences of parasites from cage-reared (Alicante) and wild (Sant Carles de la Rapita and Valencia) hosts from Spain, and one sequence from a cage-reared host from the Adriatic Sea (JX090024) [33]. From Hap_16 radiate three haplotypes from Spain (Hap_28, Hap_31, Hap_34) and one from Tunisia (Hap_55), separated by one mutation step (Figure 2). Hap_19 included eleven sequences of parasites from cage-reared (Torre Grande and Stintino) and wild lagoon (Corru S’Ittiri) hosts from Italy, and four from cage-reared (Alicante) and wild (Sant Carles de la Rapita) hosts from Spain. Hap_25 grouped five sequences from cage-reared (Orosei, Olbia, and Golfo Aranci) and wild (lagoon in Corru S’Ittiri) hosts from Italy, five sequences from cage-reared (Alicante) and wild (Sant Carles de la Rapita and Valencia) hosts from Spain, and one from a wild (Sfax) host from Tunisia. Hap_25 appeared to be separated from Hap_19 by one mutation step. The genetic distance within groups varied from 0.38% to 1.24% for samples from the Adriatic Sea and Tunisia, respectively. The genetic distance between Spanish samples and those from the Italian Sea started at 1.25% but increased to 2.54% between Tunisian samples and those from the Adriatic Sea.

The AMOVA was performed by considering the 19 populations who revealed that 43.68% of the variance was related to differences among individuals, and 47.13% was related to differences among groups (Table 3). The genetic diversity was considerably high (FST = 0.56320, *p* < 0.00001). The remaining fixation indices of this analysis were also significant, those being FSC = 0.17375 and FCT = 0.47134. Pairwise FST values between groups were all significant (Table 4). For *L. echeneis* populations isolated from *S. aurata*, FST values usually increased with the geographical distance, and the highest ones were detected between the Adriatic Sea and Spain populations (FST = 0.69968), and the Adriatic Sea and Tunisia populations (FST = 0.69397). When considering the monogeneans from the Adriatic Sea and the rest of the Mediterranean Sea, AMOVA attributed 35.39%, 16.63%, and 47.99% of the total variation to differences within populations, among populations within groups, and among groups (Table 5). Fixation indices were significant with an FST value (higher than the first run FST, 0.64612). The last AMOVA, performed on all *L. echeneis* samples except for those from the Adriatic Sea, attributed 65.84%, 12.38%, and 21.78% of the total variation to differences within populations, among populations within groups, and among groups (Table 6). The genetic diversity was high (FST = 0.34158, *p* < 0.00001). The remaining fixation indices of this analysis were also significant, those being FSC = 0.15827 and FCT = 0.21778. The rate of gene flow (Nm) increased from 0.12–2.13 when adding the sequences of parasites of *S. aurata* collected in this study and the reference ones.

**Figure 3:**
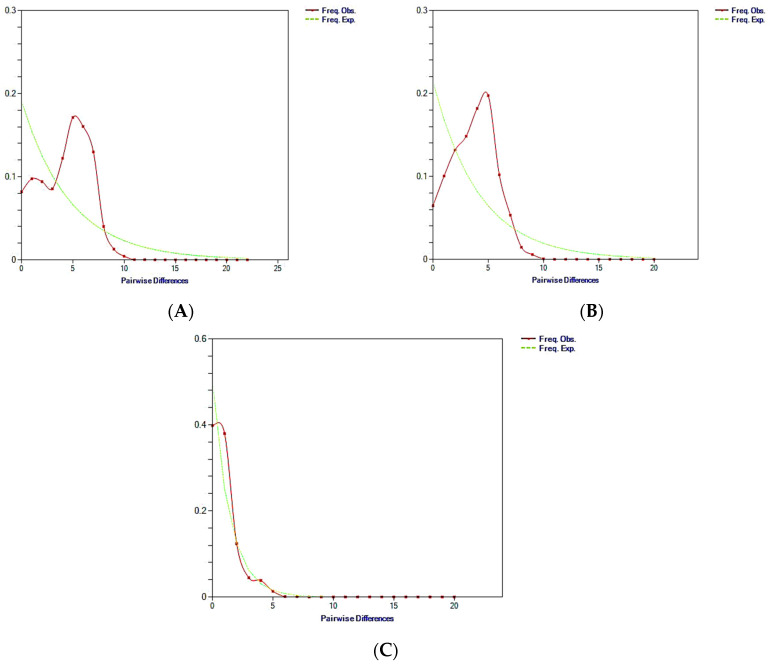
Mismatch distribution graphs for *Lamellodiscus echeneis* from the gills of *Sparus aurata* from the Mediterranean Sea. (**A**) all populations from the Mediterranean Sea; (**B**) the populations from the Mediterranean Sea excluding those from the Adriatic Sea; (**C**) the Adriatic Sea samples. The x axis displays the number of pairwise differences and the y axis displays the frequency of the pairwise comparisons. The observed frequencies are indicated by the red dotted line. The frequency expected under the hypothesis of population expansion model is represented by the continuous green line.

Historical demographic expansions were also analysed using frequency distributions of pairwise differences between sequences (all mismatch distributions are shown in Figure 3). The mismatch distribution plot for all populations from the Mediterranean Sea (Tunisia, Italy, Spain, the Adriatic Sea, and the Gulf of Lion) showed a multimodal and ragged shape (Figure 3A). Excluding the Adriatic Sea population, the mismatch distribution plot for the remaining populations also showed a multimodal and ragged pattern (Figure 3B). However, the mismatch distribution plot for all Adriatic Sea samples showed a skewed unimodal distribution, typically associated with a recent expansion or bottleneck (Figure 3C).

### 3.2. Phylogenetic Analysis

**Figure 4:**
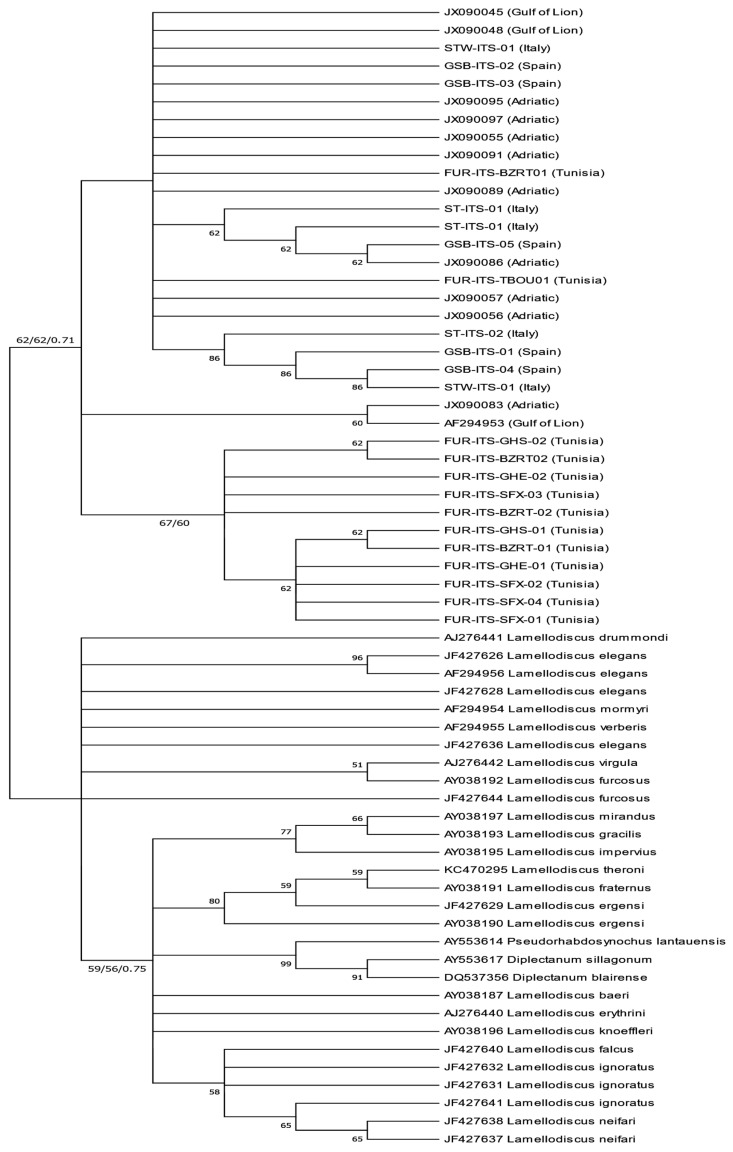
Neighbour-Joining, Maximum Likelihood, and Bayesian phylogenetic tree of *Lamellodiscus echeneis* from the gills of *Sparus aurata* from the Mediterranean Sea, based on partial ITS ribosomal DNA gene (872 bp). Terminal nodes within the *L. echeneis* clade represent the studied populations. Support values for each node are the bootstrap values of NJ, ML (**right**) posterior probability of BI (**left**). Only nodal support values > 50% are shown.

**Figure 5:**
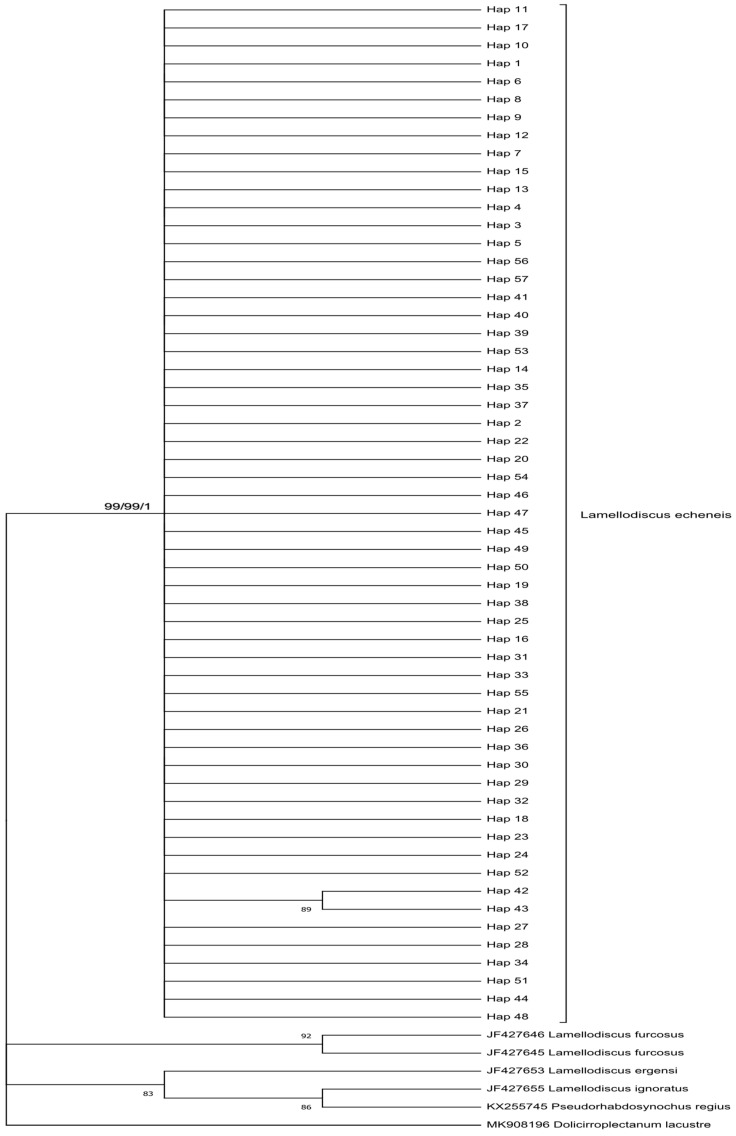
Neighbour-Joining, Maximum Likelihood, and Bayesian phylogenetic tree of *Lamellodiscus echeneis* from the gills of *Sparus aurata* from the Mediterranean Sea, based on partial COI sequences (266 bp). Terminal nodes within the *L. echeneis* clade represent the studied populations. Support values for each node are the bootstrap value of NJ, ML (**right**) posterior probability of BI (**left**). Only nodal support values > 50% are shown.

An 872 bp partial fragment of the ITS rDNA was sequenced for 23 specimens of *L. echeneis* (Tunisia (*n* = 13), Italy (*n* = 5), and Spain (*n* = 5)) from cage-reared and wild *S. aurata* specimens collected across different sampling years. These sequences were submitted in GenBank under the accession numbers PP914032-54. The most suitable substitution model for the ITS dataset was GTR + I + G for Neighbour-Joining (NJ), Maximum Likelihood (ML), and Bayesian analysis. Phylogenetic trees were constructed using NJ, ML, and BI, and revealed similar topologies. The entire 872 bp fragment remained conserved among *L. echeneis* specimens from cage-reared and wild *S. aurata* from various localities, forming a unique clade supported by intermediated bootstrap values (Bootstrap Support [BS = 62/62]) and a high posterior probability (Posterior Probability [PP = 0.71]) (Figure 4). Furthermore, within this clade, ITS sequences formed a highly supported monophyletic group with *L. echeneis* sequences from the Adriatic Sea (JX090055-JX090057, JX090083, JX090086, JX090089, JX090091, JX090095, JX090097) and the Gulf of Lion (accession numbers: JX090045, JX090048, AF294953). In all, 266 bp partial COI sequences were obtained for 97 specimens of *L. echeneis* from Tunisia, Italy, and Spain (Table 1). The best substitution model for the COI dataset was the GTR + I + G model. The obtained phylogenetic trees (NJ, ML, and BI) revealed similar topologies. All samples were grouped into the main clade, highly supported by the bootstrap values (99/99) and posterior probability (1), and corresponding to *L. echeneis* from several Mediterranean localities, including specimens from the Adriatic Sea and the Gulf of Lion (Figure 5).

## 4. Discussion

This study is the first to analyse the population genetics of the monogenean *L. echeneis*, isolated from both wild and cage-reared gilthead seabream populations in the Northern and Southern regions of the Mediterranean Sea. A comparison of the *L. echeneis* ITS region of rDNA exhibited high blast scores (99–100%) compared to previously published sequences from the *Adriatic Sea* and the Gulf of Lion, further corroborating the spread of only one species in the Mediterranean Sea. Thus, the phylogenetic analysis using the partial fragment of the ITS rDNA was consistent with previous studies on *L. echeneis* [12,33]. COI haplotyping based on a sequence alignment of a partial COI (266 bp) of populations from Tunisia, Italy, and Spain allowed us to compare their potential genetic similarity and their phylogenetic relationships to other strains from other localities (Adriatic Sea and Gulf of Lion). The 57 COI haplotypes found in this study reflected a high genetic diversity of this species within the surveyed area. Forty out of the fifty-seven haplotypes were new, whereas seventeen of them coincided with those previously described by Mladineo et al. [33] from three populations off of the Adriatic Sea and the Gulf of Lion. Among the haplotypes of the species, seventeen were exclusive to Tunisia, eight to Italy, twelve to Spain, three to the Gulf of Lion, twelve to the Adriatic Sea, and four were shared (one between the Adriatic Sea and Italian populations; one between the Adriatic Sea and Spanish populations; one between the Italian and Spanish populations; and one between the Tunisian, Italian, and Spanish populations). The haplotypes shared between wild and farmed hosts provided an insight into the population genetic variation of *L. echeneis*, confirming the potential for cross-infection between wild and farmed hosts, as reported by Mladineo et al. [33]. Concerning the pathogen transmission from wild to cage-reared fish (and vice versa), the risk of parasite transfer is plausible since infected wild fish usually stay around fish farms, and the direct life cycle of the parasite allows the fish-to-fish transmission by means of the ciliated swimming larva (oncomiracidium) [33,43,44]. Moreover, escaped fish may further transmit pathogens to other cages, as well as to wild fish [45]. The COI sequence divergence among haplotypes ranged from 0.38% to 1.24%, which is in line with what was found in other monogeneans, such as *Haliotrema aurigae* (Yamaguti, 1968) Plaisance, Bouamer & Morand, 2004 (from 0.17% to 0.70%), and *Euryhaliotrematoides grandis* (Mizelle and Kritsky, 1969) (from 0.17% to 7.99%) on the butterflyfish species *Chaetodon vagabundus* Linnaeus, 1758, and *Chaetodon auriga* Forsskål, 1775, respectively [46]. The star-like haplotypes network suggested a recent population expansion of *L. echeneis*. This is in line with the observed high haplotype diversity (Hd = 0.918) and low nucleotide diversity (Pi = 0.01595), indicating a rapid demographic expansion [47]. Monogeneans often exhibit low levels of genetic differentiation among geographic regions [48,49], and the population genetic structure of *L. echeneis* in this study appeared to conform to this pattern. This is in agreement with the low levels of the intraspecific variation in some species of *Polylabroides* (1.89%), microcotylids (4.5%), and mazocraeids (5.6%) [50,51,52]. *Lamellodiscus echeneis* is a species extensively distributed and frequently encountered across the Mediterranean Sea, and its large population size may account for the high levels of haplotype diversity observed in this region. In fact, a large population size and high nucleotide-mutation rates may be the main contributors to high genetic diversity [53,54,55]. In the present study, the mismatch distribution plot for all populations from the Mediterranean Sea displayed a multimodal and ragged pattern, suggesting a demographic equilibrium or a stable population [56]. This contrasted the observed star-like haplotypes network and population genetic indices, indicating a demographic expansion. Generally, a multimodal mismatch distribution implies diminishing population size or structure [57], while a unimodal mismatch distribution indicates recent demographic expansion or bottleneck [58]. Conversely, a ragged distribution indicates widespread lineage [59,60,61]. However, the observed multimodal mismatch distributions might also result from population sub-structuring and mutation rate heterogeneity [62,63]. The mismatch distribution analysis performed on Mediterranean populations (excluding the Adriatic Sea) also displayed multimodal characteristics, suggesting population equilibrium, possibly due to a colonisation event involving random haplotype lineages. Therefore, the observed multimodal pattern is likely attributable to distinct haplogroups, as observed in the haplotype network, rather than demographic stability. According to AMOVA analyses based on partial COI sequences, the observed pattern of genetic variability attributed 43.68% and 35.39% of the total genetic variation to variability within populations, and 47.13% and 47.99% to variation among groups, when considering samples from the Mediterranean Sea, the Adriatic Sea, and the rest of Mediterranean Sea, respectively. Fixation indices were significant, with an FST value higher than the first run (FST = 0.64612). This pattern of genetic variability may be related to the existence of a barrier between monogenean populations of the Adriatic Sea and the rest of the Mediterranean Sea. Populations located toward the distribution limits exhibited discrete biological units due to reduced migration rates, and consequently increased genetic drift [33,64,65,66]. In the present study, the mismatch distribution plot for all samples from the Adriatic Sea exhibited a skewed unimodal distribution, typically associated with a recent expansion or bottleneck [58,59,60,61], which may point to a sub-regional division and differentiation of parasite populations within the Mediterranean Sea (Adriatic Sea versus other Mediterranean regions). The highest level of genetic differentiation found among populations from the Adriatic Sea and the rest of the Mediterranean also confirmed the pattern (FST pairwise: 0.61146–0.69968). It is suggested that the semi-enclosed Adriatic Basin could represent one of the most defined phylogeographic Mediterranean regions. Reduced gene flow between the Adriatic Sea and the rest of the Mediterranean Sea has been documented for several other parasite species, such as *Sparicotyle chrysophrii* (Van Beneden and Hesse, 1863), Mamaev, 1984; *Ceratothoa oestroides* (Risso, 1827); *Aggregata octopiana* (Schneider, 1875) Frenzel, 1885 and *A. eberthi* (Labbé, 1895) Léger & Duboscq, 1906 [50,67]. This result was confirmed by excluding the Adriatic population from the AMOVA. In this case, most of the variation occurred within populations (65.84%), while 12.38% and 21.78% of the total variation occurred among populations within groups and among groups, respectively. Based on COI sequences, phylogenetic analyses, grouping all samples into the main clade corresponding to *L. echeneis* from several localities revealed no clades that could be associated with any geographic pattern. Therefore, the general pattern suggested for *L. echeneis* populations in the Mediterranean is the absence of a local genetic structure, combined with a high rate of gene flow, although the Adriatic populations represent an exception according to their local structuring compared to the rest of localities.

Li et al. [51] suggested that the genetic homogeneity of the mazocraeid *Mazocraeoides gonialosae,* Tripathi, 1959, was related to the dispersal of eggs and larvae, as this species lays single eggs without filaments and appendages in the water column, and they hypothesised that the eggs and larvae of *M. gonialosae* were capable of passively drifting for a considerable distance in ocean currents. However, this is not applicable for several species of monogeneans (including *L. echeneis*), which produce eggs with filaments that become entangled in the host’s gills and hatch after a few days. Moreover, the dispersal effect of swimming larvae may also be low, as oncomiracidia can survive for only a few hours, e.g., 3–5 h for *Pseudodactylogyrus anguillae* (Yin and Sproston, 1948); 4–8 h for *Pseudorhabdosynochus lantauensis* (Beverley-Burton & Suriano, 1981) Kritsky & Beverley-Burton, 1986; 50–58 h for *Dawestrema cycloancistrium* (Price and Nowlin, 1967); and 2–52 h for *S. chrysophrii* [68,69,70,71]. For parasites, the opportunities for spread are determined not only by their own dispersal abilities, but also by the vagility and characteristics of their hosts [72]. Host-specificity is a key property of the dispersal ability of ectoparasites [73]; since *L. echeneis* is a specific parasite of *S. aurata*, its dispersal may be related only to this host species. Many studies have investigated the genetic structure of wild and farmed *S. aurata* populations across the Mediterranean Sea based on allozyme, microsatellite markers, and DNA sequences [74,75,76,77,78]. Maroso et al. [79] found differentiation between wild Atlantic and Mediterranean gilthead seabream populations, as well as within Mediterranean populations. Specifically, their analyses suggested a weak subdivision of gilthead seabream into four major genetic clusters (Atlantic, Western Mediterranean, Ionian/Adriatic Seas, and the Aegean Sea). However, the findings of Villanueva et al. [78], which were based on a large number of SNP analyses and diverse sample sources, corroborated the divergence between Atlantic and Mediterranean populations, but without supporting differentiation within the Mediterranean Sea. Indeed, numerous studies have suggested a lack of genetic structure of gilthead seabream when using different types of genetic markers, also attributed to the widening of its distribution related to global warming and recurring escape events from fish farms [75,76,78,80,81]. Therefore, the lack of a clear genetic structure of *L. echeneis* based on mtDNA COI can be attributed to significant gene flow between populations, favoured by the host dispersion potential within the Mediterranean Sea [78].

## 5. Conclusions

In conclusion, this study provides insight into the population genetic variation of *L. echeneis*, showing an absence of genetic structure and a high level of gene flow through Tunisian, Italian, and Spanish localities. The observed patterns of genetic variation within and between *L. echeneis* populations are most likely caused by a recent demographic expansion. On the other hand, the haplotypes shared between wild and farmed hosts confirmed the potential for cross-infection, providing molecular evidence for pathogen transfer. Further studies in other areas would be useful to deepen the knowledge of the population structure of *L. echeneis*. Analysing other diplectanid species and their hosts could reveal potential general patterns, shedding light on the genetic dynamics of *L. echeneis,* and revealing if observed patterns in the Mediterranean Sea apply to other Dactylogyridae species as well.

## Figures and Tables

**Table 1:** List of the examined specimens of *Lamellodiscus echeneis* from the gills of wild and cage-reared *Sparus aurata*. Acronym: geographic acronyms and initialisms. N hosts: number of hosts; N parasites: total number of parasites; n-COI: parasites for population genetic analysis; n-ITS: parasites for phylogenetic analysis.

Localities (Acronym *)	Year	N Hosts	N Parasites	n-COI	n-ITS
Tunisia Wild					
Bizerte (BZRT)	2023	4	27	6	4
Ghar El Melh (GHS)	2023	6	57	6	2
Sfax (SFX)	2023	10	25	7	4
Cage-reared					
Ghar El Melh (GHE)	2023	10	94	6	2
Teboulba (TBOU)	2023	10	27	7	1
Djerba (DJE)	2023	10	94	6	
Sardinia (Italy) Wild					
Corru s’Ittiri (CI)	2010	5	310	6	
Stintino (STW)	2023	9	74	5	2
Cage-reared					
Golfo Aranci (4F/4C)	2022	5	262	5	
Olbia (OL)	2005	5	174	2	
Orosei (OR)	2005	5	99	2	
Stintino (ST)	2019–2023	10	197	6	2
Torre Grande (TGG)	2023	5	70	6	1
Spain Wild					
La Rapita (GSB01-15)	2023	40	15	15	5
Valencia (GSB16-GSB17)	2023	11	2	2	
Cage-reared					
Alicante (GSB18-GSB27)	2023	15	16	10	
Total		160	1543	97	23

* See Supplementary Materials Table S1.

**Table 2:** Standard population genetics statistics of *Lamellodiscus echeneis* from the gills of *Sparus aurata* from the Mediterranean Sea based on partial COI: number of sequences (n), number of haplotypes (k), number of polymorphic sites (PS), haplotype diversity (Hd), and nucleotide diversity (Pi).

Population	n	k	PS	Hd	Pi
Adriatic Sea	51	0.99216	17	0.60157	0.00373
Gulf of Lion	3	2.00000	3	1.00000	0.00752
Italy	32	3.04637	10	0.84879	0.01145
Spain	27	2.74074	15	0.92877	0.01030
Tunisia	38	3.19488	19	0.79943	0.01201
Mediterranean Sea *	100	3.67899	28	0.93535	0.01383

* All Mediterranean localities except for the Adriatic Sea.

**Table 3:** Analysis of molecular variance (AMOVA) of *Lamellodiscus echeneis* from the gills of *Sparus aurata* from the Mediterranean Sea. Percentage of variation explained by different hierarchical levels for partial COI. Degrees of freedom (d.f.). * *p* < 0.001.

Source of Variation	d.f.	Sum of Squares	Variance Components	% Variation	*p*-Value
Among groups	4	137.551	1.14936 Va	47.13	*
Among populations within groups	14	35.424	0.22399 Vb	9.19	*
Within populations	125	133.143	1.06514 Vc	43.68	*
Total	143	306.118	2.43849		

Fixation Indices: FSC: 0.17375; FST: 0.56320; FCT: 0.47134.

**Table 4:** Pairwise FST results between populations (lower diagonal) and associated significance indications (upper diagonal). * *p* < 0.001.

	Adriatic Sea	Gulf of Lion	Italy	Spain	Tunisia
Adriatic Sea	-	*	*	*	*
Gulf of Lion	0.68421	-	*	*	*
Italy	0.61146	0.38483	-	*	*
Spain	0.69968	0.46178	0.10421	-	*
Tunisia	0.69397	0.48502	0.24031	0.27467	-

**Table 5:** Analysis of molecular variance (AMOVA) of *Lamellodiscus echeneis* from the gills of *Sparus aurata* from the Adriatic Sea and the rest of Mediterranean Sea. Percentage of variation explained by different hierarchical levels for partial COI. Degrees of freedom (d.f.). * *p* < 0.001.

Source of Variation	d.f.	Sum of Squares	Variance Components	% Variation	*p*-Value
Among groups	1	99.394	1.44432 Va	47.99	*
Among populations within groups	17	73.581	0.50040 Vb	16.63	*
Within populations	125	133.143	1.06514 Vc	35.39	*
Total	143	306.118	3.00987		

Fixation Indices: FSC: 0.31963; FST: 0.64612; FCT: 0.47986.

**Table 6:** Analysis of molecular variance (AMOVA) of *Lamellodiscus echeneis* from the gills of *Sparus aurata* from the Mediterranean Sea, excepting those from the Adriatic Sea. Percentage of variation explained by different hierarchical levels for partial COI. Degrees of freedom (d.f.). * *p* < 0.001.

Source of Variation	d.f.	Sum of Squares	Variance Components	% Variation	*p*-Value
Among groups	3	38.156	0.43374 Va	21.78	*
Among populations within groups	13	35.111	0.24657 Vb	12.38	*
Within populations	83	108.843	1.31136 Vc	65.84	*
Total	99	182.110	1.99166		

Fixation Indices: FSC: 0.15827; FST: 0.34158; FCT: 0.21778.

## Data Availability

Sequence data have been deposited in GenBank under the accession numbers PP892317-73 and PP914032-54.

## Supplementary Materials

The following supporting information can be downloaded at: https://www.mdpi.com/article/10.3390/ani14182653/s1, Table S1: List of *Lamellodiscus echeneis* sequences used in the phylogenetic analyses. Date, host, locality, GenBank accession numbers for COI and ITS, and the reference are also included. Table S2: Observed COI mitochondrial haplotypes of *Lamellodiscus echeneis* from the Mediterranean Sea used in this study, with their frequency, code, host, and locality.
