# Supplementary material for: Assessment of the Genetic Diversity of the Monogenean Gill Parasite Lamellodiscus echeneis (Monogenea) Infecting Wild and Cage-Reared Populations of Sparus aurata (Teleostei) from the Mediterranean Sea
Source: Animals (Basel). 2024 Sep 12;14(18):2653. doi: 10.3390/ani14182653 (PMC11429135; doi:10.3390/ani14182653)
Supplement: Supplementary file 1 [file animals-14-02653-s001.zip › animals-3154466-supplementary.pdf]

Supplementary Materials Table S1: List of *Lamellodiscus echeneis* sequences used in the phylogenetic analyses. Date, host, locality, GenBank accession numbers for COI and ITS, and reference are also included.

	Sample	DATE	Host		Locality	Accession number COI	Accession number ITS	Reference
1	TTG-05	*2023*	*S. aurata*	Cage	Italy	PP892317		This study
2	TTG-03	*2023*	*S. aurata*	Cage	Italy	PP892317		This study
3	4F-02	*2022*	*S. aurata*	Cage	Italy	PP892317		This study
4	GSB-18	*2023*	*S. aurata*	Cage	Spain	PP892332		This study
5	GSB-09	*2023*	*S. aurata*	Wild	Spain	PP892332		This study
6	GSB-08	*2023*	*S. aurata*	Wild	Spain	PP892332		This study
7	TTG-06	*2023*	*S. aurata*	Cage	Italy	PP892334		This study
8	TTG-04	*2023*	*S. aurata*	Cage	Italy	PP892335		This study
9	TTG-02	*2023*	*S. aurata*	Cage	Italy	PP892335		This study
10	ST-04	*2019*	*S. aurata*	Cage	Italy	PP892335		This study
11	ST-03	*2019*	*S. aurata*	Cage	Italy	PP892335		This study
12	ST-02	*2019*	*S. aurata*	Cage	Italy	PP892335	PP914046	This study
13	ST-01	*2019*	*S. aurata*	Cage	Italy	PP892335	PP914045	This study
14	GSB-19	*2023*	*S. aurata*	Cage	Spain	PP892335		This study
15	GSB-12	*2023*	*S. aurata*	Wild	Spain	PP892335		This study
16	GSB-07	*2023*	*S. aurata*	Wild	Spain	PP892335		This study
17	GSB-05	*2023*	*S. aurata*	Wild	Spain	PP892335	PP914052	This study
18	CI-04	*2010*	*S. aurata*	Wild	Italy	PP892335		This study
19	CI-05	*2019*	*S. aurata*	Wild	Italy	PP892335		This study
20	CI-03	*2019*	*S. aurata*	Wild	Italy	PP892335		This study
21	CI-01	*2019*	*S. aurata*	Wild	Italy	PP892335		This study
22	CI-02	*2019*	*S. aurata*	Wild	Italy	PP892335		This study
23	TTG-01	*2023*	*S. aurata*	Cage	Italy	PP892336	PP914050	This study
24	OL-01	*2005*	*S. aurata*	Cage	Italy	PP892336		This study
25	STW-05	*2023*	*S. aurata*	Wild	Italy	PP892337		This study
26	STW-04	*2023*	*S. aurata*	Wild	Italy	PP892338		This study
27	STW-03	*2023*	*S. aurata*	Wild	Italy	PP892339		This study
28	STW-02	*2023*	*S. aurata*	Wild	Italy	PP892339	PP914054	This study
29	ST-06	*2019*	*S. aurata*	Cage	Italy	PP892339		This study
30	ST-05	*2019*	*S. aurata*	Cage	Italy	PP892339		This study
31	STW-01	*2023*	*S. aurata*	Wild	Italy	PP892340	PP914053	This study
32	OR-02	*2005*	*S. aurata*	Cage	Italy	PP892341		This study
33	OR-01	*2005*	*S. aurata*	Cage	Italy	PP892341		This study
34	OL-02	*2005*	*S. aurata*	Cage	Italy	PP892341		This study
35	GSB-26	*2023*	*S. aurata*	Cage	Spain	PP892341		This study
36	GSB-23	*2023*	*S. aurata*	Cage	Spain	PP892341		This study
37	GSB-22	*2023*	*S. aurata*	Cage	Spain	PP892341		This study
38	GSB-13	*2023*	*S. aurata*	Wild	Spain	PP892341		This study
39	GSB-11	*2023*	*S. aurata*	Wild	Spain	PP892341		This study
40	FUR-SFX	*2023*	*S. aurata*	Wild	Tunisia	PP892341	PP914039	This study
41	CI-06	*2010*	*S. aurata*	Wild	Italy	PP892341		This study
42	4F-01	*2022*	*S. aurata*	Cage	Italy	PP892341		This study
43	GSB-27	*2023*	*S. aurata*	Cage	Spain	PP892342		This study
44	GSB-25	*2023*	*S. aurata*	Cage	Spain	PP892342		This study
45	GSB-21	*2023*	*S. aurata*	Cage	Spain	PP892342		This study
46	GSB-16	*2023*	*S. aurata*	Wild	Spain	PP892342		This study
47	GSB-24	*2023*	*S. aurata*	Cage	Spain	PP892343		This study
48	GSB-20	*2023*	*S. aurata*	Cage	Spain	PP892344		This study
49	GSB-17	*2023*	*S. aurata*	Wild	Spain	PP892345		This study
50	GSB-15	*2023*	*S. aurata*	Wild	Spain	PP892346		This study
51	GSB-14	*2023*	*S. aurata*	Wild	Spain	PP892347		This study
52	GSB-10	*2023*	*S. aurata*	Wild	Spain	PP892348		This study
53	GSB-06	*2023*	*S. aurata*	Wild	Spain	PP892349		This study
54	GSB-04	*2023*	*S. aurata*	Wild	Spain	PP892350	PP914051	This study
55	GSB-03	*2023*	*S. aurata*	Wild	Spain	PP892351	PP914047	This study
56	GSB-02	*2023*	*S. aurata*	Wild	Spain	PP892352	PP914049	This study
57	GSB-01	*2023*	*S. aurata*	Wild	Spain	PP892353	PP914048	This study
58	FUR-GHS	*2023*	*S. aurata*	Wild	Tunisia	PP892354	PP914036	This study
59	FUR-GHS	*2023*	*S. aurata*	Wild	Tunisia	PP892355	PP914035	This study

60	FUR-GHS	*2023*	*S. aurata*	Wild	Tunisia	PP892356		This study
61	FUR-GHS	*2023*	*S. aurata*	Wild	Tunisia	PP892357		This study
62	FUR-BZRT03	*2023*	*S. aurata*	Wild	Tunisia	PP892357	PP914040	This study
63	FUR-GHS	*2023*	*S. aurata*	Wild	Tunisia	PP892358		This study
64	FUR-BZRT06	*2023*	*S. aurata*	Wild	Tunisia	PP892358	PP914041	This study
65	FUR-GHS	*2023*	*S. aurata*	Wild	Tunisia	PP892359		This study
66	FUR-GHE	*2023*	*S. aurata*	Cage	Tunisia	PP892360	PP914032	This study
67	FUR-GHE	*2023*	*S. aurata*	Cage	Tunisia	PP892361	PP914042	This study
68	FUR-GHE	*2023*	*S. aurata*	Cage	Tunisia	PP892362		This study
69	FUR-GHE	*2023*	*S. aurata*	Cage	Tunisia	PP892362		This study
70	FUR-GHE	*2023*	*S. aurata*	Cage	Tunisia	PP892362		This study
71	FUR-DJE	*2023*	*S. aurata*	Cage	Tunisia	PP892362		This study
72	FUR-DJE	*2023*	*S. aurata*	Cage	Tunisia	PP892362		This study
73	FUR-TBOU	*2023*	*S. aurata*	Cage	Tunisia	PP892362		This study
74	FUR-TBOU	*2023*	*S. aurata*	Cage	Tunisia	PP892362		This study
75	FUR-TBOU	*2023*	*S. aurata*	Cage	Tunisia	PP892362		This study
76	FUR-TBOU	*2023*	*S. aurata*	Cage	Tunisia	PP892362		This study
77	FUR-TBOU	*2023*	*S. aurata*	Cage	Tunisia	PP892362		This study
78	FUR-SFX	*2023*	*S. aurata*	*Wild*	Tunisia	PP892362	PP914033	This study
79	FUR-SFX	*2023*	*S. aurata*	*Wild*	Tunisia	PP892362	PP914034	This study
80	FUR-SFX	*2023*	*S. aurata*	*Wild*	Tunisia	PP892362	PP914038	This study
81	FUR-SFX	*2023*	*S. aurata*	*Wild*	Tunisia	PP892362		This study
82	FUR-SFX	*2023*	*S. aurata*	*Wild*	Tunisia	PP892362		This study
83	FUR-SFX	*2023*	*S. aurata*	*Wild*	Tunisia	PP892362		This study
84	FUR-BZRT05	*2023*	*S. aurata*	*Wild*	Tunisia	PP892362	PP914043	This study
85	FUR-GHE	*2023*	*S. aurata*	Cage	Tunisia	PP892363		This study
86	FUR-DJE	*2023*	*S. aurata*	Cage	Tunisia	PP892363		This study
87	FUR-DJE	*2023*	*S. aurata*	Cage	Tunisia	PP892363		This study
88	FUR-DJE	*2023*	*S. aurata*	Cage	Tunisia	PP892364		This study
89	FUR-DJE	*2023*	*S. aurata*	Cage	Tunisia	PP892365		This study
90	FUR-TBOU	*2023*	*S. aurata*	Cage	Tunisia	PP892366	PP914044	This study
91	FUR-TBOU	*2023*	*S. aurata*	Cage	Tunisia	PP892367		This study
92	4F-TO-01	*2022*	*S. aurata*	Cage	Italy	PP892368		This study
93	FUR-BZRT04	*2023*	*S. aurata*	*Wild*	Tunisia	PP892369		This study
94	FUR-BZRT02	*2023*	*S. aurata*	*Wild*	Tunisia	PP892370	PP914037	This study
95	FUR-BZRT01	*2023*	*S. aurata*	*Wild*	Tunisia	PP892371		This study
96	4C-02	*2022*	*S. aurata*	Cage	Italy	PP892372		This study
97	4C-01	*2022*	*S. aurata*	*Cage*	Italy	PP892373		This study
98	C1a	*2010*	*S. aurata*	*Cage*	*Adriatic Sea*	JX089988	JX090055	Mladineo et al. 2013
99	C1b	*2010*	*S. aurata*	*Cage*	*Adriatic Sea*	JX089989	JX090056	Mladineo et al. 2013
100	C3a	*2010*	*S. aurata*	*Cage*	*Adriatic Sea*	JX089990		Mladineo et al. 2013
101	C3b	*2010*	*S. aurata*	*Cage*	*Adriatic Sea*	JX089991	JX090057	Mladineo et al. 2013
102	C4a	*2010*	*S. aurata*	*Cage*	*Adriatic Sea*	JX089992		Mladineo et al. 2013
103	C4b	*2010*	*S. aurata*	*Cage*	*Adriatic Sea*	JX089993		Mladineo et al. 2013
104	C6a	*2010*	*S. aurata*	*Cage*	*Adriatic Sea*	JX089994		Mladineo et al. 2013
105	C6b	*2010*	*S. aurata*	*Cage*	*Adriatic Sea*	JX089995		Mladineo et al. 2013
106	C17a	*2010*	*S. aurata*	*Cage*	*Adriatic Sea*	JX089996		Mladineo et al. 2013
107	C5a	*2010*	*S. aurata*	*Cage*	*Adriatic Sea*	JX090001		Mladineo et al. 2013
108	C5b	*2010*	*S. aurata*	*Cage*	*Adriatic Sea*	JX090002		Mladineo et al. 2013
109	C13c	*2010*	*S. aurata*	*Cage*	*Adriatic Sea*	JX090005		Mladineo et al. 2013
110	C10a	*2010*	*S. aurata*	*Cage*	*Adriatic Sea*	JX090006		Mladineo et al. 2013
111	C13d	*2010*	*S. aurata*	*Cage*	*Adriatic Sea*	JX090008		Mladineo et al. 2013
112	Cf4c	*2010*	*S. aurata*	*Cage*	*Adriatic Sea*	JX090013		Mladineo et al. 2013
113	Cf5b	*2010*	*S. aurata*	*Cage*	*Adriatic Sea*	JX090016		Mladineo et al. 2013
114	Cf1a	*2010*	*S. aurata*	*Cage*	*Adriatic Sea*	JX090018		Mladineo et al. 2013
115	Cf2c	*2010*	*S. aurata*	*Cage*	*Adriatic Sea*	JX090021		Mladineo et al. 2013
116	Cf6b	*2010*	*S. aurata*	*Cage*	*Adriatic Sea*	JX090022		Mladineo et al. 2013
117	Cf7a	*2010*	*S. aurata*	*Cage*	*Adriatic Sea*	JX090023	JX090083	Mladineo et al. 2013
118	Cf7b	*2010*	*S. aurata*	*Cage*	*Adriatic Sea*	JX090026	JX090086	Mladineo et al. 2013
119	Cf2b	*2010*	*S. aurata*	*Cage*	*Adriatic Sea*	JX090027		Mladineo et al. 2013
120	Cf7d	*2010*	*S. aurata*	*Cage*	*Adriatic Sea*	JX090028		Mladineo et al. 2013
121	Cf1c	*2010*	*S. aurata*	*Cage*	*Adriatic Sea*	JX090030		Mladineo et al. 2013
122	W1a	*2010*	*S. aurata*	*Wild*	*Adriatic Sea*	JX090031	JX090091	Mladineo et al. 2013
123	W1b	*2010*	*S. aurata*	*Wild*	*Adriatic Sea*	JX090032		Mladineo et al. 2013
124	W1c	*2010*	*S. aurata*	*Wild*	*Adriatic Sea*	JX090033		Mladineo et al. 2013
125	W3	*2010*	*S. aurata*	*Wild*	*Adriatic Sea*	JX090035	JX090095	Mladineo et al. 2013
126	W4a	*2010*	*S. aurata*	*Wild*	*Adriatic Sea*	JX090036		Mladineo et al. 2013
127	W4b	*2010*	*S. aurata*	*Wild*	*Adriatic Sea*	JX090037	JX090097	Mladineo et al. 2013

128	W5a	*2010*	*S. aurata*	*Wild*	*Adriatic Sea*	JX090038		Mladineo et al. 2013
129	W6a	*2010*	*S. aurata*	*Wild*	*Adriatic Sea*	JX090040		Mladineo et al. 2013
130	C17b	*2010*	*S. aurata*	*Cage*	*Adriatic Sea*	JX089997		Mladineo et al. 2013
131	FW1a	*2010*	*S. aurata*	*Wild*	Gulf of Lion	JX089998		Mladineo et al. 2013
132	FW1b	*2010*	*S. aurata*	*Wild*	Gulf of Lion	JX089999	JX090048	Mladineo et al. 2013
133	FW2	*2010*	*S. aurata*	*Wild*	Gulf of Lion	JX090000		Mladineo et al. 2013
134	C8	*2010*	*S. aurata*	*Cage*	*Adriatic Sea*	JX090003		Mladineo et al. 2013
135	C13b	*2010*	*S. aurata*	*Cage*	*Adriatic Sea*	JX090004		Mladineo et al. 2013
136	C10b	*2010*	*S. aurata*	*Cage*	*Adriatic Sea*	JX090007		Mladineo et al. 2013
137	Cf4a	*2010*	*S. aurata*	*Cage*	*Adriatic Sea*	JX090011		Mladineo et al. 2013
138	Cf4b	*2010*	*S. aurata*	*Cage*	*Adriatic Sea*	JX090012		Mladineo et al. 2013
139	W2	*2010*	*S. aurata*	*Wild*	*Adriatic Sea*	JX090034		Mladineo et al. 2013
140	W7	*2010*	*S. aurata*	*Wild*	*Adriatic Sea*	JX090009		Mladineo et al. 2013
141	W8	*2010*	*S. aurata*	*Wild*	*Adriatic Sea*	JX090010		Mladineo et al. 2013
142	Cf5a	*2010*	*S. aurata*	*Cage*	*Adriatic Sea*	JX090014		Mladineo et al. 2013
143	Cf4d	*2010*	*S. aurata*	*Cage*	*Adriatic Sea*	JX090015		Mladineo et al. 2013
144	Cf5c	*2010*	*S. aurata*	*Cage*	*Adriatic Sea*	JX090017		Mladineo et al. 2013
145	Cf6c	*2010*	*S. aurata*	*Cage*	*Adriatic Sea*	JX090025		Mladineo et al. 2013
146	W5b	*2010*	*S. aurata*	*Wild*	*Adriatic Sea*	JX090039		Mladineo et al. 2013
147	W6b	*2010*	*S. aurata*	*Wild*	*Adriatic Sea*	JX090041		Mladineo et al. 2013
148	Cf2a	*2010*	*S. aurata*	*Cage*	*Adriatic Sea*	JX090019		Mladineo et al. 2013
149	Cf3a	*2010*	*S. aurata*	*Cage*	*Adriatic Sea*	JX090020		Mladineo et al. 2013
150	Cf7c	*2010*	*S. aurata*	*Cage*	*Adriatic Sea*	JX090024		Mladineo et al. 2013
151	Cf2d	*2010*	*S. aurata*	*Cage*	*Adriatic Sea*	JX090029	JX090089	Mladineo et al. 2013
152	FW3a	*2010*	*S. aurata*	*Wild*	*Gulf of Lion*	-	JX090045	Mladineo et al. 2013
153	AF294953	*2001*	*S. aurata*	*Wild*	*Gulf of Lion*	-	AF294953	Desdevises 2001

Supplementary Materials Table S2 Observed COI mitochondrial haplotypes of *Lamellodiscus echeneis* from the Mediterranean Sea used in this study, with their frequency, code, host and locality.

**Haplotype**	**Feq**	**Sample code**	**Host**	**Localities**	**Host origin**
Hap_1	35	[JX089988 JX089989 JX089990 JX089991 JX089992 JX089993 JX089994 JX089995 JX089996 JX090001 JX090002 JX090005 JX090006 JX090008 JX090013 JX090016 JX090018 JX090021 JX090022 JX090023 JX090026 JX090027 JX090028 JX090030 JX090031 JX090032 JX090033 JX090035 JX090036 JX090037 JX090038 JX090040 TTG-05 TTG-03 4F-02]	*S. aurata*	Adriatic Sea/Italy	Cage/ Wild
Hap_2	1	[JX089997]	*S. aurata*	Adriatic Sea	Cage
Hap_3	1	[JX089998]	*S. aurata*	Gulf of Lion	Wild
Hap_4	1	[JX089999]	*S. aurata*	Gulf of Lion	Wild
Hap_5	1	[JX090000]	*S. aurata*	Gulf of Lion	Wild
Hap_6	1	[JX090003]	*S. aurata*	Adriatic Sea	Cage
Hap_7	1	[JX090004]	*S. aurata*	Adriatic Sea	Cage
Hap_8	4	[JX090007 JX090011 JX090012 JX090034]	*S. aurata*	Adriatic Sea	Cage/ Wild
Hap_9	1	[JX090009]	*S. aurata*	Adriatic Sea	Wild
Hap_10	1	[JX090010]	*S. aurata*	Adriatic Sea	Wild
Hap_11	1	[JX090014]	*S. aurata*	Adriatic Sea	Cage
Hap_12	1	[JX090015]	*S. aurata*	Adriatic Sea	Cage
Hap_13	4	[JX090017 JX090025 JX090039 JX090041]	*S. aurata*	Adriatic Sea	Cage/ Wild
Hap_14	1	[JX090019]	*S. aurata*	Adriatic Sea	Cage
Hap_15	1	[JX090020]	*S. aurata*	Adriatic Sea	Cage
Hap_16	4	[JX090024 GSB-18 GSB-09 GSB-08]	*S. aurata*	Adriatic Sea/Spain	Cage/Wild
Hap_17	1	[JX090029]	*S. aurata*	Adriatic Sea	Cage
Hap_18	1	[TTG-06]	*S. aurata*	Italy	Cage

Hap_19	15	[TTG-04 TTG-02 ST-04 ST-03 ST-02 ST-01 GSB-19 GSB-12 GSB-07 GSB-05 CI-04 CI-05 CI-03 CI-01 CI-02]	*S. aurata*	Italy/Spain	Cage/Wild
Hap_20	2	[TTG-01 OL-01]	*S. aurata*	Italy	Cage
Hap_21	1	[STW-05]	*S. aurata*	Italy	Wild
Hap_22	1	[STW-04]	*S. aurata*	Italy	Wild
Hap_23	4	[STW-03 STW-02 ST-06 ST-05]	*S. aurata*	Italy	Cage/Wild
Hap_24	1	[STW-01]	*S. aurata*	Italy	Wild
Hap_25	11	[OR-02 OR-01 OL-02 GSB-26 GSB-23 GSB-22 GSB-13 GSB-11 FUR-SFX CI-06 4F-01]	*S. aurata*	Italy/Spain/Tunisia	Cage/Wild
Hap_26	4	[GSB-27 GSB-25 GSB-21 GSB-16]	*S. aurata*	Spain	Cage/Wild
Hap_27	1	[GSB-24]	*S. aurata*	Spain	Cage
Hap_28	1	[GSB-20]	*S. aurata*	Spain	Cage
Hap_29	1	[GSB-17]	*S. aurata*	Spain	Wild
Hap_30	1	[GSB-15]	*S. aurata*	Spain	Wild
Hap_31	1	[GSB-14]	*S. aurata*	Spain	Wild
Hap_32	1	[GSB-10]	*S. aurata*	Spain	Wild
Hap_33	1	[GSB-06]	*S. aurata*	Spain	Wild
Hap_34	1	[GSB-04]	*S. aurata*	Spain	Wild
Hap_35	1	[GSB-03]	*S. aurata*	Spain	Wild
Hap_36	1	[GSB-02]	*S. aurata*	Spain	Wild
Hap_37	1	[GSB-01]	*S. aurata*	Spain	Wild
Hap_38	1	[FUR-GHS]	*S. aurata*	Tunisia	Wild
Hap_39	1	[FUR-GHS]	*S. aurata*	Tunisia	Wild
Hap_40	1	[FUR-GHS]	*S. aurata*	Tunisia	Wild
Hap_41	2	[FUR-GHS FUR-BZRT03]	*S. aurata*	Tunisia	Wild
Hap_42	2	[FUR-GHS FUR-BZRT06]	*S. aurata*	Tunisia	Wild
Hap_43	1	[FUR-GHS]	*S. aurata*	Tunisia	Wild
Hap_44	1	[FUR-GHE]	*S. aurata*	Tunisia	Cage
Hap_45	1	[FUR-GHE]	*S. aurata*	Tunisia	Cage
Hap_46	17	[FUR-GHE FUR-GHE FUR-GHE FUR-DJE FUR-DJE FUR-TBOU FUR-TBOU FUR-TBOU FUR-TBOU FUR-TBOU FUR-SFX FUR-SFX FUR-SFX FUR-SFX FUR-SFX FUR-SFX FUR-BZRT05]	*S. aurata*	Tunisia	Cage/ Wild
Hap_47	3	[FUR-GHE FUR-DJE FUR-DJE]	*S. aurata*	Tunisia	Cage
Hap_48	1	[FUR-DJE]	*S. aurata*	Tunisia	Cage
Hap_49	1	[FUR-DJE]	*S. aurata*	Tunisia	Cage
Hap_50	1	[FUR-TBOU]	*S. aurata*	Tunisia	Cage
Hap_51	1	[FUR-TBOU]	*S. aurata*	Tunisia	Cage
Hap_52	1	[4F-TO-01]	*S. aurata*	Italy	Cage
Hap_53	1	[FUR-BZRT04]	*S. aurata*	Tunisia	Wild
Hap_54	1	[FUR-BZRT02]	*S. aurata*	Tunisia	Wild
Hap_55	1	[FUR-BZRT01]	*S. aurata*	Tunisia	Wild
Hap_56	1	[4C-02]	*S. aurata*	Italy	Cage
Hap_57	1	[4C-01]	*S. aurata*	Italy	Cage
